# Development of a Short-Term Embolic Agent Based on Cilastatin for Articular Microvessels

**DOI:** 10.3390/medicina60091538

**Published:** 2024-09-20

**Authors:** Hyun Jin Kim, Areum Jeon, Eun Kyung Kang, Wen An, So Jung Lim, Kyu Chul Shin, Dong Hun Shin, Inyoung Hwang, Ju Seop Kang

**Affiliations:** 1 Department of Pharmacology, College of Medicine, Hanyang University, Seoul 04736, Republic of Korea; hope0211@hanyang.ac.kr (H.J.K.);; 2 Exercise Physiology Lab, Department of Physical Education, Graduate School, Korea University, Seoul 02841, Republic of Korea; 3 Cheil Orthopedic Hospital, 726 Yeongdong-daero, Gangnam-gu, Seoul 06075, Republic of Korea; 4 S&J Core Inc., 9 Yeongdong-daero 106-gil, Gangnam-gu, Seoul 06170, Republic of Korea; 5 Department of Clinical Pharmacology and Therapeutics, Hanyang University Seoul Hospital, Seoul 04736, Republic of Korea

**Keywords:** cilastatin, embolic agent, D-mannitol, short-term embolic efficacy

## Abstract

*Background and Objectives*: This study aimed to develop an embolic agent with short-term embolic effects using cilastatin as the basic material. *Materials and Methods*: The particle size distribution of 25 mg cilastatin-based short-term embolic agents was evaluated microscopically under three different mixing conditions. A total of thirty-six healthy male Sprague Dawley rats were divided into four groups. Each group of six rats was injected once into the tail artery with 0.4 mL each of (A) Cilastatin + D-Mannitol Mixture, (B) Iohexol, (C) Prepenem, and (D) embolization promoter (EGgel). *Results*: A visual inspection of the tail appearance of rats in each group was performed at 0, 3, 7, 15, and 21 days. At weeks 1 and 3, three rats per group were euthanized, and histopathological analyses were performed on the specimens obtained from each group. No significant differences were observed on day 7, but mild inflammation was observed in Group (D) on day 15. Histopathological inflammation scoring of tail central artery embolization was performed using a six-point scale (from 0 = absent to 5 = marked inflammation). Three groups were formed consisting of six male New Zealand white rabbits each: control, positive control, and test groups. The control group received an Iohexol injection (rabbits: 0.8 mL). The positive control and experimental groups were injected with prepenem and cilastatin/D-mannitol compound, respectively (0.8 mL), and vascular angiography was performed. The order of occlusion progression after embolization was as follows: test group, positive control group, and control group. *Conclusions*: We developed a cilastatin/D-mannitol compound that exhibits characteristics of short-term embolization by utilizing the pharmacokinetic properties of cilastatin and the crystalline material D-mannitol. We evaluated its particle size distribution microscopically, conducted histopathological evaluation including inflammation via animal experiments, and assessed the embolization effect.

## 1. Introduction

In osteoarthritis (OA), there is an increase in angiogenesis within various joint components, leading to structural advancement, tissue heterogeneity, and pain initiation [1,2,3,4]. Modulating angiogenesis and nerve growth presents a promising avenue for OA symptom management [5,6,7]. Future approaches might entail the use of angiogenesis inhibitors to slow down the progression of joint damage, potentially offering pain relief [8]. Recent research suggests support for further investigation into anti-angiogenic therapies for OA, as treatment including angiogenesis inhibitors has shown potential in alleviating pain-related responses [9]. However, further research is required to understand the precise association between ossification and OA symptoms [10]. Strategies like suppressing inflammation, decreasing nerve density, and preserving osteochondral junctions can directly alleviate patient symptoms, with the anticipation that angiogenesis inhibition could modulate these mechanisms to alleviate pain [3,11].

Inhibiting angiogenesis can target vascular cells directly or indirectly suppress inflammation. Strategies encompass disrupting the breakdown of the extracellular matrix and the formation of osteochondral channels crucial for vascular growth [12,13]. Direct-acting angiogenesis inhibitors like VEGF blockers and receptor tyrosine kinase inhibitors are employed to curb tumor growth by focusing on angiogenesis [14,15]. Given the similarities in vascular formation mechanisms between osteoarthritis (OA) and tumors, these medications might also be efficacious in OA management [2,11,16].

Consequently, tackling moderate OA remains a formidable challenge [17]. Ongoing research aims to inhibit angiogenesis for OA treatment, with transcatheter arterial embolization (TAE) in the knee joint potentially aiding in pain relief and functional restoration [18,19,20].

Therapeutic embolization entails intentionally administering occlusive agents into blood vessels to impede blood flow [21,22]. The selection of embolic agents hinges on desired clinical outcomes and the characteristics of the embolic agent [23]. A comprehensive understanding of the properties of various agents is paramount for clinical efficacy [24]. While autologous blood clots were historically prevalent, contemporary embolic agents are available in diverse forms such as liquids, particles, coils, detachable plugs, and balloons [23,25].

D-Mannitol, a sugar alcohol, finds extensive use in both pharmaceutical formulations and food products [26,27]. Within the pharmaceutical realm, it serves as a vital excipient, being employed in various dosage forms for its role as a diluent and flavor enhancer [27]. Due to its nature as atypical small molecule polyhydroxy compound, D-mannitol has long been recognized for its polymorphism, existing in three distinct forms: α, β, and δ [27,28,29]. The α and β forms exhibit needle-like morphology, while the δ form appears smooth and rod-like [27,30]. Furthermore, variations in crystal forms induce changes in D-mannitol’s particle size [31,32]. Therefore, harnessing the polymorphic crystallization of D-mannitol can underscore its efficacy as a particle-forming embolic agent.

Cilastatin (CS) inhibits the enzymatic conversion of Leukotriene(LT)D4 to LTE4 and disrupts the metabolism of thienamycin β-lactam antibiotics by renal dehydropeptidase [33,34]. It is eliminated via renal excretion without undergoing metabolic transformations within the body [35]. Imipenem, a substrate of renal dehydropeptidase, is predominantly prescribed for renal infections [36]. To preserve its therapeutic efficacy, it is co-administered with CS to impede its metabolism [36,37]. This combined medication, marketed as Primaxin by Merck, serves as the impetus for the present investigation [38]. This initiative is grounded in the minimal metabolic activity and low pharmacological toxicity associated with CS [39]. Hence, the objective of this investigation is to formulate a locally acting embolic substance utilizing Cilastatin and evaluate its efficacy as a temporary embolic agent via both in vitro and in vivo experiments.

## 2. Materials and Methods

### 2.1. Materials

Cilastatin sodium salt (CS) was obtained from Sigma-Aldrich (St. Louis, MO, USA). Prepenem (imipenem-cilastatin) was obtained from JW Pharm. Co., Ltd. (Gwacheon, Republic of Korea). D-Mannitol was procured from Daejung Chemicals & Metals Co., Ltd. (Seoul, Republic of Korea). EGgel S Plus was purchased from ENGAIN Co., LTD. (Hwaseong, Republic of Korea). Omnipaque (Iohexol) was purchased from GE Healthcare (Marlborough, MA, USA). All other reagents were of analytical grade. 

### 2.2. Preparation and Application of Embolic Material

Cilastatin/D-Mannitol Compound consisted of Cilastatin 25 mg and D-Mannitol 230 mg. The embolic solution of Cilastatin/D-Mannitol was prepared in 1 mL of distilled water (DW). Prepenem 500 mg (Cilastatin Sodium 500 mg, Imipenem Monohydrate 500 mg) was prepared in 20 mL of DW. This embolic solution was injected once into the tail mid-ventral artery for toxicity testing.

For angiographic analysis, a formulation consisting of 25 mg Cilastatin, 230 mg D-Mannitol, and 1 mL contrast agent was prepared as an embolic solution. The contrast agent was mixed with 2 mL of Iohexol in a vial containing 510 mg of Cilastatin/D-Mannitol mixture. Subsequently, the compounded solution was drawn into a 5 mL syringe and gently administered using a 2.5 mL syringe connected via a three-way stopcock. This solution was injected through a microcatheter inserted into the ear artery until the observation of blood flow cessation. All solutions were prepared immediately prior to injection, and the lids tightly closed.

### 2.3. Animals Study

For the toxicity tests, male Sprague Dawley (SD) rats aged 12 weeks (350 g ± 30 g) were purchased from JA BIO (Suwon, Republic of Korea). The experimental rats were accommodated in the Laboratory Animal Research Facility situated at Hanyang University’s Medical Research Support Center. The experimental protocol followed ethical standards sanctioned by the Institutional Animal Care and Use Committee (IACUC) at the Medical Research Support Center, Hanyang University (HY-2022-0249 A). All rats underwent an acclimatization period for a minimum of seven days prior to the start of the experiment, during which they were maintained at a temperature of 23 ± 1 °C with a 12 h light/dark cycle. During both the acclimatization and experimental phases, the rats were provided with unlimited access to standard rat chow and water. The rats were divided into four groups of six rats each for acute and chronic toxicity studies, with reference to previous research protocol [23,40,41,42]: (A) Cilastatin + D-Mannitol Mixture (25 mg + 230 mg/1 mL (DW)), (B) Iohexol, (C) Prepenem (Cilastatin Sodium 25 mg + Imipenem Monohydrate 25 mg/1 mL (DW)), and (D) embolization promoter (EGgel) treatment group. Each embolic solution (0.4 mL) was administered once into the rat caudal artery and three rats each were sacrificed for histological examination on days 7 and 21 after administration. 

To evaluate the embolotherapy induced by tail mid-ventral artery embolization, three groups of six healthy male New Zealand white rabbits were formed to test the intra-arterial distribution of cilastatin formulations in the left ear of the rabbits [43]. The control group was administered iohexol (contrast agent), the positive control group was administered prepenem (Cilastatin Sodium 25 mg + Imipenem Monohydrate 25 mg/2 mL (iohexol)), and the test group was administered cilastatin/D-Mannitol (25 mg + 230 mg/1 mL (iohexol)); 0.8 mL of each drug formulation was injected into the left ear of two rabbits.

### 2.4. Morphological Analysis 

The compound’s morphology analysis was performed using the method of Hirakawa et al. [40]. The compound of Cilastatin/D-Mannitol combination was filtered through 0.45 um membrane (HA type, Millipore, Burlington, MA, USA) immediately after dissolution, and the membrane was gently rinsed with tertiary distilled water and dried using a desiccator at room temperature.

The compound’s image were examined using an optical microscope and scanning electron microscopy (SEM) with an accelerating voltage of 15 kV (APREO FEI, Houston, TX, USA). Prior to SEM observation, the samples were mounted on a carbon-taped stub and coated with platinum for 80 s under vacuum conditions.

### 2.5. Histopathologic Analysis 

Samples from rat tails were immersed in 4% paraformaldehyde for fixation before being embedded in paraffin to ensure long-term preservation. Tissue sections with a thickness of 4 μm were prepared and subsequently stained using either hematoxylin and eosin (H&E) [44] or Masson’s trichrome (MT) [45]. Light microscopy examination was conducted for all groups, and images representing the typical histological profile were reviewed. Histological inflammation of the tail mid-ventral artery was assessed using a simple scoring system (0, absent; 1, minimal; 2, mild; 3, moderate; 4, severe; and 5, marked inflammation). Collagen deposition in the rat tail mid-ventral artery is confirmed by Masson’s trichrome staining.

### 2.6. Angiographic Analysis

The prepared compound was administered into the left ear artery of New Zealand white rabbits. The embolization process of the test substance was visualized using C-Arm imaging and assessed based on distribution time. All solutions were prepared immediately prior to injection, and the lids were tightly closed. Each rabbit was imaged in a recumbent position on a single plane C-arm machine (ARCADIS Varic, Simens Healthcare GmBH, Erlangen, Germany).

## 3. Results

### 3.1. Preparation and Comparison of Cilastatin-Based Short-Term Embolization Material

**Figure 1:**
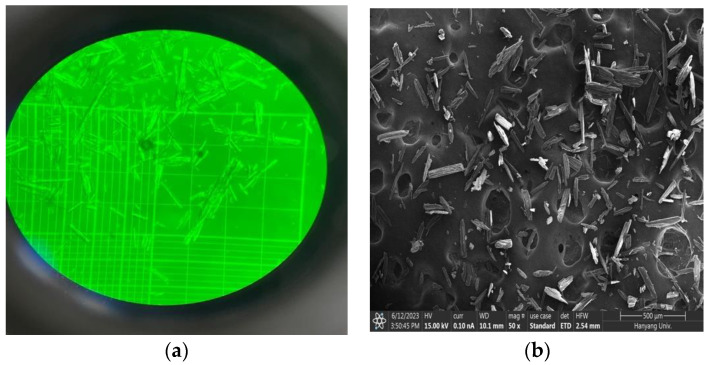
Particle size and distribution in compound; the image is an optical microscope photograph (**a**) of the Cilastatin/D-Mannitol combination, while the image on the right is taken with a scanning electron microscope (SEM) (**b**) The particle length is approximately 96.0 ± 30.0 μm.

The development of Cilastatin/D-Mannitol combination involved using 25 mg of Cilastatin and 230 mg of D-Mannitol in 1 mL of tertiary distilled water (Table 1). Optical and scanning electron microscopes were used for particle size measurement (Figure 1). The shape and size of particles are crystalline with an average diameter of approximately 96.0 ± 30.0 μm. All microscopy images were captured immediately after manufacturing.

### 3.2. Local and Systemic Toxicity Evaluation Following Tail Vein Injection of Test Substance

**Figure 2:**
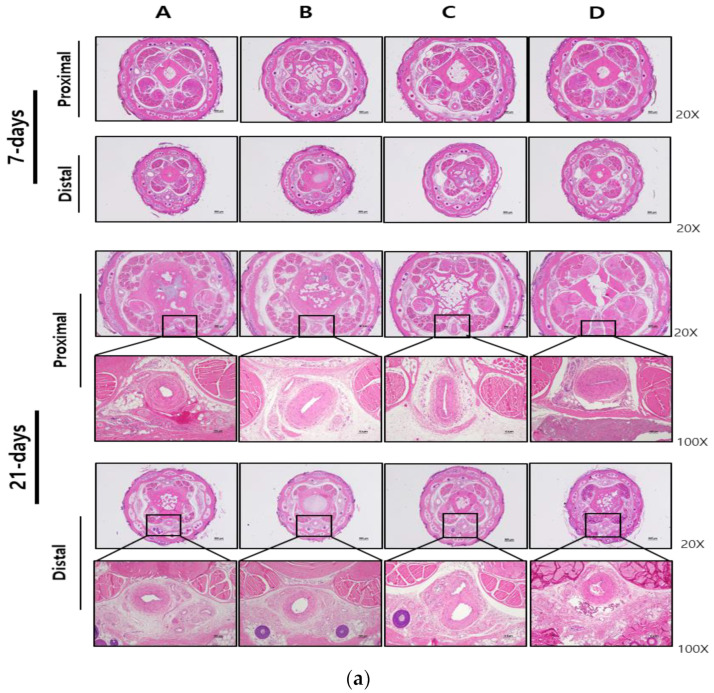

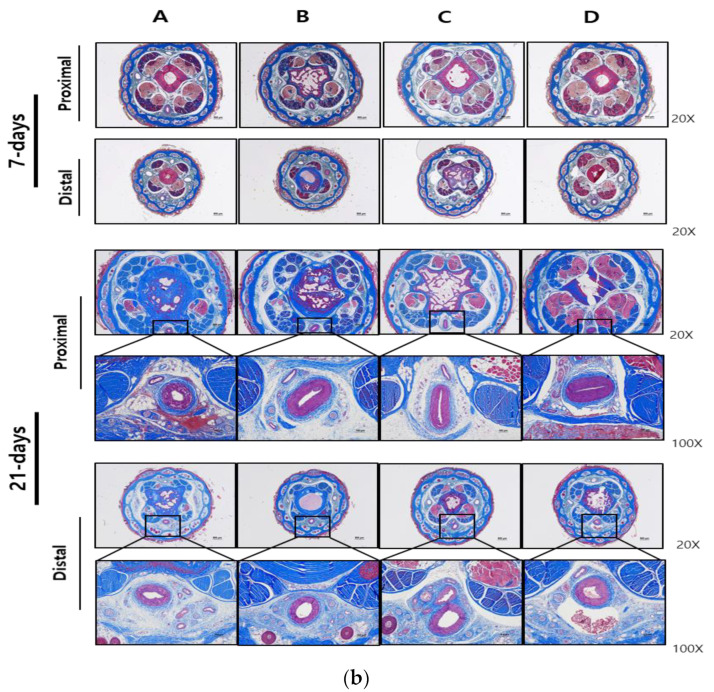
Histopathological observations of proximal and distal sections of the middle part of the rat tail stained with (**a**) Hematoxylin and eosin and (**b**) Masson’s trichrome, 7 days (**upper** panel) and 21 days (**lower** panel) after injection of the embolic substances. Group A: Cilastatin + D-Mannitol Mixture, Group B: Iohexol, Group C: Prepenem, Group D: EGgel. Magnifications: 20× (scale bar is 500 µm), 100× (scale bar is 100 µm).

The rats were divided into four groups (six rats in each group) and injected once with three types of embolic agents and one contrast agent. The rats were observed at designated time intervals (0, 3, 7, 15, and 21 days) for a total of 5 days, and three animals were sacrificed on the day 7 and three animals on day 21 after the administration for histological examination. After the sacrifice, the rat tail was divided into three parts, and the middle segment of the tail was prepared as a tissue blocks, which was fixed in 4% (*w*/*v*) paraformaldehyde for 48 h and then embedded in paraffin. Tissue sections were made in transverse sections of the proximal and distal portions of the tail block, including the mid-ventral artery, and stained with H&E and Masson’s trichrome. Representative pathological findings of the lesion sites in the tissues of each group were observed under a microscope, and the chronic thrombotic effect (toxicity) was evaluated based on the observations. Figure 2 showed the histopathological evaluation performed each group on days 7 and 21. Histological inflammation of the tail mid-ventral artery was assessed using a simple scoring system and with reference to a previously described method [44]. Histological analysis revealed that no inflammation or stenosis due to embolic agents in the mid-ventral artery at the proximal and distal portions of the tail was observed on day 7. On day 21, mild inflammation in the distal part and stenosis in the proximal part of the mid-ventral artery was observed in the control group, intravascular thrombolytic agent EGgel (D). In contrast, no vascular inflammation or stenosis was observed in the other groups.

Table 2 showed that local and systemic toxicity was assessed by capturing necrotic changes occurring in the rat tail and including histological inflammation scores of the rat tail mid-ventral artery. 

D group showed a tendency of inflammatory signs from the 15 days onwards, and clear toxic pathological findings were observed in the histopathological examination on the 21 days. No toxicity was observed in the experimental agents, iohexol, and prepenem groups. 

Acute (7 days) and chronic (21 days) toxicity was not observed in the test agent, iohexol, and prepenem drugs based on appearance and histopathological findings. However, only the control drug, EGgel, showed chronic toxicity as evidenced by the appearance of the tail lesion after 15 days and moderate inflammation in histological analysis on day 21.

### 3.3. Angiographic Results

**Figure 3:**
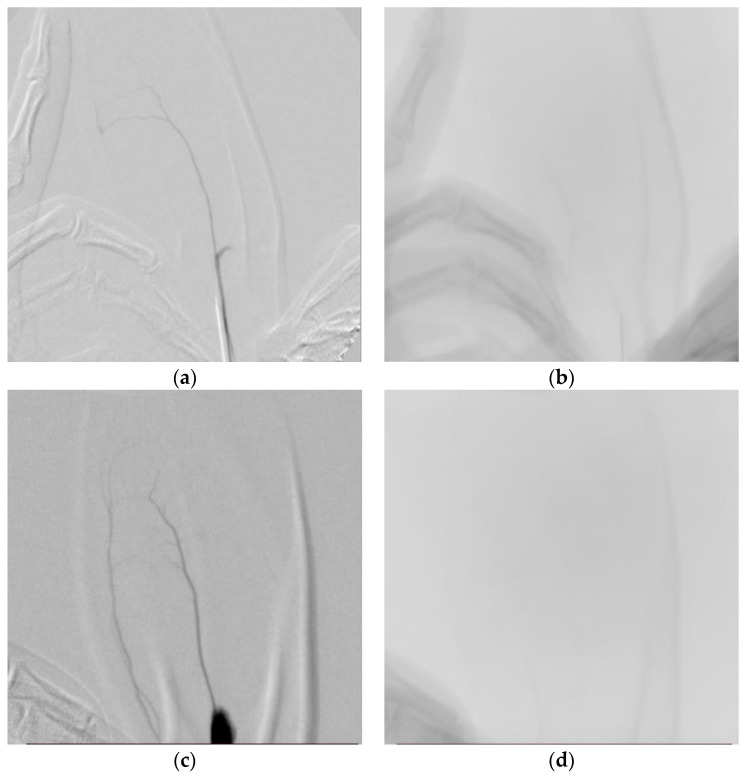

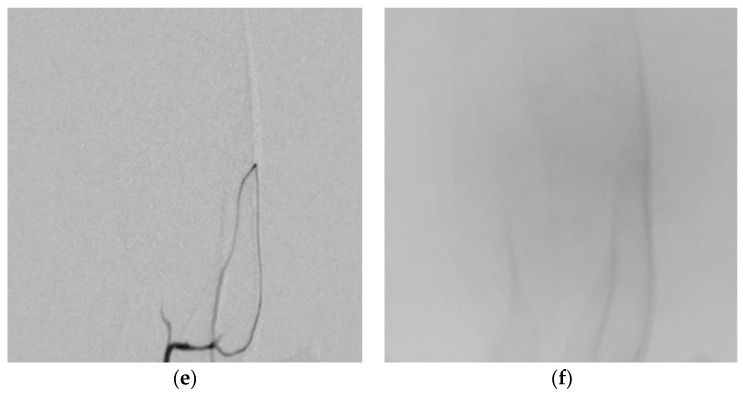
Angiographic analysis. The distribution pattern of Cilastatin/D-Mannitol (contrast solution) is evaluated by administering it into the ear artery of New Zealand white rabbits and imaging it with micro-CT (C-Arm) to assess the distribution time. The figures show (**a**) 10 s after iohexol administration, (**b**) 120 s after iohexol administration, (**c**) 55 s after prepenem administration, (**d**) 120 s after prepenem administration, (**e**) 40 s after Cilastatin/D-Mannitol administration, and (**f**) 120 s after Cilastatin/D-Mannitol administration.

The embolotherapy effect of the embolic agent was evaluated using rabbits because the rabbit’s ear artery is wide and highly visible. The distribution pattern of the embolic agents (contrast solution mixture) in the ear artery of New Zealand white rabbits is evaluated based on the distribution time through micro-CT (C-Arm) imaging. The rabbits were anesthetized with an appropriate intravenous injection of an anesthetic and placed on the operating table. The rabbit’s left ear hair was shaved using an electric shaver and then disinfected. After making an incision in the skin near the base of the ear, the central artery of the ear is identified. A 22G IV catheter is inserted into the ear artery of the rabbit, and the test drug suspension (0.8 mL) is gradually infused from the base of the ear through the central artery. The left and right panels of Figure 3 show results approximately 10~55 s and 120 s after test drug administration, respectively. Figure 3a,b are images after the administration of the contrast solution (iohexol); the iohexol image disappeared at 10 s and, the image was checked up to 120 s. Figure 3c,d are images after the administration of the positive control drug (prepenem), and the drug was clearly contrasted at 55 s; it was hardly visible at about 60 s. Figure 3e,f are images after the administration of the test drug (Cilastatin/D-Mannitol). The test drug began to contrast around 40 s, and by 120 s, there was no contrast at all. Thus, the test drug can be considered suitable as a temporary contrast agent that demonstrates the intended short-term contrast effect.

## 4. Discussion

This study assessed the development and efficacy of a locally acting short-acting anticoagulant based on cilastatin. Given that the pharmacokinetic properties of cilastatin suggest minimal pharmacological toxicity without undergoing metabolic processes in the body, the physical properties of cilastatin were utilized to examine the characteristics of a Cilastatin and D-Mannitol mixture as a local anticoagulant and to evaluate its potential application as an anticoagulant agent. The evaluation encompassed the analyses of particle size distribution, local toxicity assessment, and efficacy evaluation for anticoagulation procedures.

Cilastatin/D-Mannitol comprises sodium cilastatin and D-Mannitol, with sodium cilastatin being a white to pale yellow powder that readily dissolves in water and methanol. When 25 mg of Cilastatin and 230 mg of D-Mannitol were dissolved in 1 mL of contrast medium, the particle size consistently ranged within 30–100 μm despite noticeable irregularities.

For acute and chronic local toxicity assessments, observations were made on the external appearance and histopathological findings of the tail arteries of white rats. The efficacy and toxicity of arterial embolization agents are usually performed in relatively large organ arteries (such as renal arteries), but since this developed product is administered to the articular artery, it must be performed in a very small artery. Therefore, the central tail artery of a white rat was selected, and since short-term and long-term observations can be made with the naked eye, it has the advantage of ease of experimentation. In addition, since representative examples of the toxicity of embolization agents include inflammation/necrosis in the peripheral area due to physical arterial embolization, histopathological analysis was also conducted for the purpose of observing inflammation and necrosis [23,40,41,42]. EGgel, a contrast-promoting agent, exhibited inflammation from the 7th day and clear toxic pathological findings from the 21st day. Conversely, no toxicity was observed in the groups treated with Cilastatin/D-Mannitol, Iohexol, and Prepenem.

In the anticoagulation efficacy experiment, drugs were administered in the ear arteries of New Zealand white rabbits, followed by micro-CT (C-Arm) imaging to evaluate the contrast agent distribution time. The mean contrast agent residence times were approximately 10 s for iohexol, 55 s for Prepenem, and 40 s for Cilastatin/D-Mannitol. Although differences existed in residence times, they were not markedly significant. The disappearance of crystals in Prepenem and test substance solutions during mixing with contrast agents suggested reduced anticoagulation effects due to microcrystals. Future experiments are planned to separately analyze the anticoagulation effects of the anticoagulant and contrast agents.

## 5. Conclusions

The study synthesized a crystalline compound formulation with transient embolic properties using 25 mg of Cilastatin sodium salt and 230 mg of D-Mannitol. The morphology and particle size of the Cilastatin/D-Mannitol blend were analyzed via optical and electron microscopy. Histological analysis confirmed the safety of the Cilastatin/D-Mannitol compound on the central tail artery of rats. Vascular angiography tracking revealed temporary embolic effects on the rabbit’s ear artery. However, the limitation of this study is that the efficacy and toxicity were observed to a limited extent, so it is necessary to conduct the study in larger organ arteries (such as renal arteries) to confirm the embolic effect, and although this development product is an embolic agent that utilizes a physical effect, it will be necessary to evaluate the systemic toxicity. In conclusion, the developed Cilastatin/D-Mannitol mixture demonstrated reduced inflammation and short-term embolic effects, suggesting its potential as a biocompatible vascular embolic material in future applications.

## Figures and Tables

**Table 1:** Composition of the formulated Cilastatin/D-Mannitol Compound preparations; the Cilastatin/D-Mannitol combination is manufactured using 25 mg of Cilastatin and 230 mg of D-Mannitol in 1 mL of tertiary distilled water.

Component	Concentration (% *w*/*v*)
Cilastatin sodium salt	2.5
D-Mannitol	23

**Table 2:** Acute and chronic toxic effects of test substances in each group (appearance and histopathological findings).

Test Group (n = Number of Subjects)	Day 0 (n = 6)	Day 3 (n = 6)	Day 7 (n = 3)	Day 15 (n = 3)	Day 21 (n = 3)
A. Cilastatin + D-Mannitol Mixture	0	0	0 (0,0,0) ^1^	0	0 (0,0,0) ^1^
B. Iohexol	0	0	0 (0,0,0) ^1^	0	0 (0,0,0) ^1^
C. Prepenem	0	0	0 (0,0,0) ^1^	0	0 (0,0,0) ^1^
D. EGgel	0	0	0 (0,0,0) ^1^	1	3 (1,3,3) ^1^

Appearance Findings: 0 = absent; 1 = presence of 1 rat tail lesion; 3 = presence of 3 rat tail lesions. ^1^ Inflammation score: 0 = absent; 1 = minimal; 2 = mild; 3 = moderate; 4 = severe; and 5 = marked inflammation.

## Data Availability

The data and materials for this article are available from the corresponding author upon reasonable request.
